# Scene-dependent sound event detection based on multitask learning with deformable large kernel attention convolution

**DOI:** 10.1371/journal.pone.0322002

**Published:** 2025-05-09

**Authors:** Haiyue Zhang, Menglong Wu, Xichang Cai, Wenkai Liu

**Affiliations:** School of Information Science and Technology, North China University of Technology, Beijing, China; National University of Sciences and Technology NUST, PAKISTAN

## Abstract

Sound event detection (SED) and acoustic scene classification (ASC) are closely related tasks in environmental sound analysis. Given the interrelationship between sound events and scenes, some previous studies have proposed using the multitask learning (MTL) method to jointly analyze SED and ASC. However, these multitask learning methods are generally based on hard parameter-sharing, which exchange sound event and scene features only through the low-level network. Such approaches are difficult to balance the complex interrelationships between SED and ASC, and limits the feature sharing and information flow between tasks during the training. To address these challenges, this study proposes a novel multitask network based on residual multi-level feature extraction (R-MFE) framework, which aims to jointly analyze SED and ASC tasks, and utilize scene information to improve the performance of sound event detection. In addition, this study designs the D-LKAC attention module, which combines the advantages of self-attention mechanisms and convolution to capture global and local features. To further enhance SED performance, this study introduces the MS-conv module, which captures audio details from multiple dimensions. The proposed MTL method is evaluated on the TUT Acoustic Scenes 2016/2017 and TUT Sound Events 2016/2017 datasets. Experimental results indicate that our approach outperforms state-of-the-art techniques, improving the F-scores by 6.44%.

## 1. Introduction

In recent years, environmental sound analysis has received increasing attention. It has shown great potential in various application scenarios such as life recording systems [1], surveillance systems [2,3], abnormal detection systems [4], and biomonitoring systems. Sound event detection (SED) [5] and acoustic scene classification (ASC) [6] are two key tasks in the field of environmental sound analysis. SED aims to detect and categorize events in audio recordings, including “keystrokes,” “car driving,” or “alarm sounds.” The process requires identifying these events and assessing their start and end times. Meanwhile, ASC aims to distinguish and classify scene category information from audio recordings, such as typical environments like “office,” “coffee shop,” or “grocery store.” These scenes often consist of a complex mix of multiple sound events, forming an intricate and realistic acoustic environment. Recent advances in deep learning have gradually made it the mainstream method for solving complex problems like this [7–13].

In fact, acoustic scenes and sound events are closely related. Many sound events are often highly correlated with certain acoustic scenes. For example, compared to acoustic scenes like “park,” sound events such as “keyboard typing,” “mouse-clicking,” and “people talking” are more commonly in acoustic scenes like “office.” Therefore, the scene information can be used to exclude sound events that are unlikely to occur in the scene, thereby improving the accuracy of sound event detection. Given the inherent relationship between sound events and scenes, some studies have proposed using multitask learning (MTL) [14] to jointly analyze the SED and ASC tasks. Multitask learning has shown broad potential in many fields [15–20] and has gradually becoming the mainstream method for environmental sound analysis[21–25]. For instance, Liang et al.[23] introduced a novel framework based on weak supervision that combines audio tagging (AT) and SED, demonstrating strong competitiveness between tasks. Jung et al. [24] proposed a comprehensive system that simultaneously addresses tasks in AT, ASC, and SED. Furthermore, Hou et al. [25] introduced the relation matrix to the joint analysis of SED and ASC, and used probabilistic relationships to improve the performance of ASC.

Recently, Imoto et al. [26,27] utilized sound event information to enhance ASC performance within the Bayesian generative theoretical model. Afterward, neural networks based on the MTL approach [28–31] were presented to jointly analyze SED and ASC. The method combining MTL with soft scene labeling [32,33] was proposed to enhance the performance of the SED task. Unlike conventional MTL methods, the approach allows for more accurate modeling of the connections between specific scenes and events. In addition, Liang et al. [34] explored an attention-based MTL method to extract and establish shared and independent representations between scenes and events. Komatsu et al. [35] used a unidirectional conditional loss from scene to event to combine scene and event information. Tsubaki et al [36] proposed an MTL framework with weak labels to jointly analyze SED and ASC. Nada et al. [37] implemented dynamic adjustment of weights using multi-focal loss and then introduced a dynamic weight averaging strategy [38]. Subsequently, Hou et al. [39] suggested an approach for the collaborative modeling of scenes and events. This method makes full use of the connection between sound events and scenes, enabling parallel processing of SED and ASC tasks. Although the MTL techniques mentioned above can improve performance, they generally employ hard parameter-sharing strategies. This approach only uses the fundamental feature-sharing mechanism of the MTL framework to extract common features, neglecting the continuous interaction and information flow between tasks. Moreover, such methods often struggle to effectively balance the complex relationships between tasks.

To address these issues, inspired by [40,41], this study proposes a novel multitask network designed to model the relationship between acoustic scenes and sound events, while leveraging scene information to enhance SED performance. Different from earlier multitask networks based on hard-parameter sharing [42–44], the proposed MTL method distinctly separates shared and task-specific experts for SED and ASC, which reduces the interference of harmful parameters between tasks and alleviates the performance conflicts caused by task diversity in conventional multitask networks. In addition, the introduction of gated networks allows for the fusion of more abstract representations, enabling dynamic adjustment of parameter weights between shared and task-specific information. Furthermore, the network employs a multi-level feature extraction strategy to capture audio features from multiple dimensions, which further improves the efficiency of information transfer.

The main contributions of this study are as follows:

1. This study proposes a multitask network based on the R-MFE framework. The model is designed to model the relationship between acoustic scenes and sound events, and leverages scene information to enhance the performance of sound event detection.
2. This study designs a D-LKAC module. This module combines the advantages of convolution and attention mechanisms to effectively capture both global and local features in audio sequences. Moreover, compared to traditional attention mechanisms, D-LKAC can dynamically focus on adjacent time-frequency bands, capturing richer feature information.
3. To further improve the performance of the SED task, this study introduces the MS-conv module, which captures audio features more comprehensively. The proposed MTL method is compared with existing joint analysis methods for SED and ASC, and the results show that the proposed method outperforms the current state-of-the-art techniques.

This paper is structured as follows: Section 2 discusses conventional methods. Section 3 provides a detailed description of the proposed method. Section 4 discusses the experimental setup and findings. The paper concludes with Section 5.

## 2. Conventional methods

**Fig 1:**
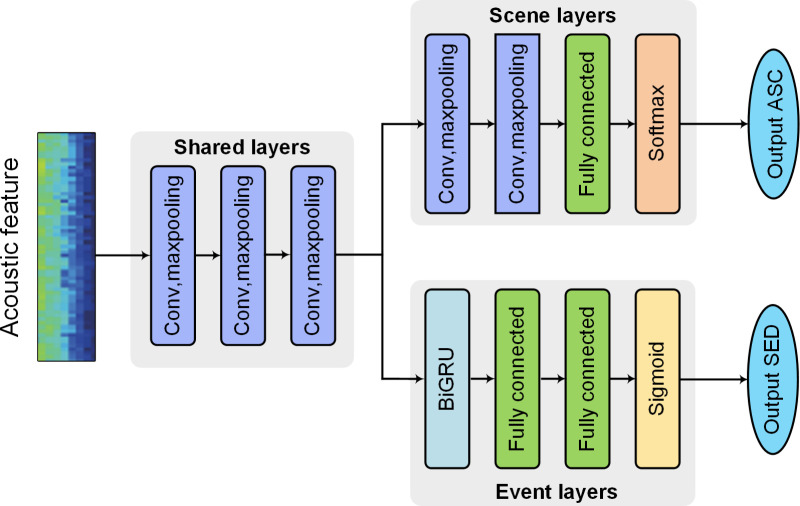
Architecture of conventional MTL-based method.

In many previous studies, SED and ASC are typically analyzed separately. However, sound events and scenes are closely linked and often co-occur, as shown in Table 1. Therefore, acoustic scene information will be beneficial in sound event detection. Based on this idea, some researchers have proposed joint analyses of SED and ASC [29–32] using the MTL, as shown in Fig 1. The architecture of these methods includes shared layers and independent branch networks for each task.

**Table 1:** The presence/absence relationship of sound events and scenes.

	(object) banging	(object) impact	(object) rustling	(object) snapping	(object) squeaking	bird singing	brakes squeaking	breathing	car	children	cupboard	cutlery	dishes	drawer	fan	glass jingling	keyboard typing	large vehicle	mouse clicking	mouse wheeling	people talking	people walking	washing dishes	Water tap running	wind blowing
City center	0	0	0	0	0	1	0	1	1	0	0	0	0	0	0	0	0	1	0	0	1	1	0	0	0
Home	0	1	1	0	0	0	0	0	0	1	1	1	1	1	0	1	0	0	0	0	0	1	1	1	0
Office	0	1	0	1	0	0	1	0	0	0	1	0	0	0	1	0	1	0	1	1	1	1	0	0	0
Residential area	1	0	1	0	1	0	0	1	1	0	0	0	0	0	0	0	0	1	0	0	1	1	0	0	1

The shared layer uses a convolutional neural network (CNN), while the task-specific branches for event detection and scene classification employ bidirectional gated recurrent unit (BiGRU) and CNN, respectively.

The loss function in the conventional MTL method usually utilizes a weighted sum approach to train multi-task models for SED and ASC. This study adopts the same approach. For the ASC task, the model parameters are optimized using the cross-entropy (CE) loss function. The loss function $L_{\text{ASC}}$ is defined as follows:


$$L_{ASC}=-\sum_{n=1}^{N}\left\{s_{n}\log(y_{n})\right\}$$ (1)

where $y_{n}$ is the network output, *N* is the number of acoustic scene categories, and *s*_*n*_ represents the target scene labels.

On the other hand, due to the possible overlapping of sound events on the time axis, this study uses the binary cross entropy (BCE) loss function to train model parameters in multi-label classification. The loss function $L_{\text{SED }}$ is expressed as:


$$\begin{array}{cc} L_{SED} & =-\sum_{t=1}^{T}\left\{z_{t}\log(y_{t})+(1+z_{t})\log(1-y_{t})\right\}\\ & =-\sum_{t=1}^{T}\sum_{m=1}^{M}\left\{z_{t,m}\log(y_{t,m})+(1-z_{t,m})\log(1-y_{t,m})\right\} \end{array}$$ (2)

where $y_{t,m}$ represents the network output, $z_{t,m}$ corresponds to the target event labels, *T* denotes the number of time frames, and *M* is the count of sound event categories.

In this study, the loss functions of SED and ASC are linearly combined with constant weights ${\;\lambda }_{ASC}$ and $\lambda_{SED}$. During the training phase, the weights of the two loss functions are optimized to improve classification accuracy. The joint loss function *L(θ)* is represented as follows:


$$L\left(\theta \right)=\lambda_{ASC}L_{\text{ASC}}\left(\theta \right)+\lambda_{SED}L_{\text{SED}}\left(\theta \right)$$ (3)

## 3. Proposed method

**Fig 2:**
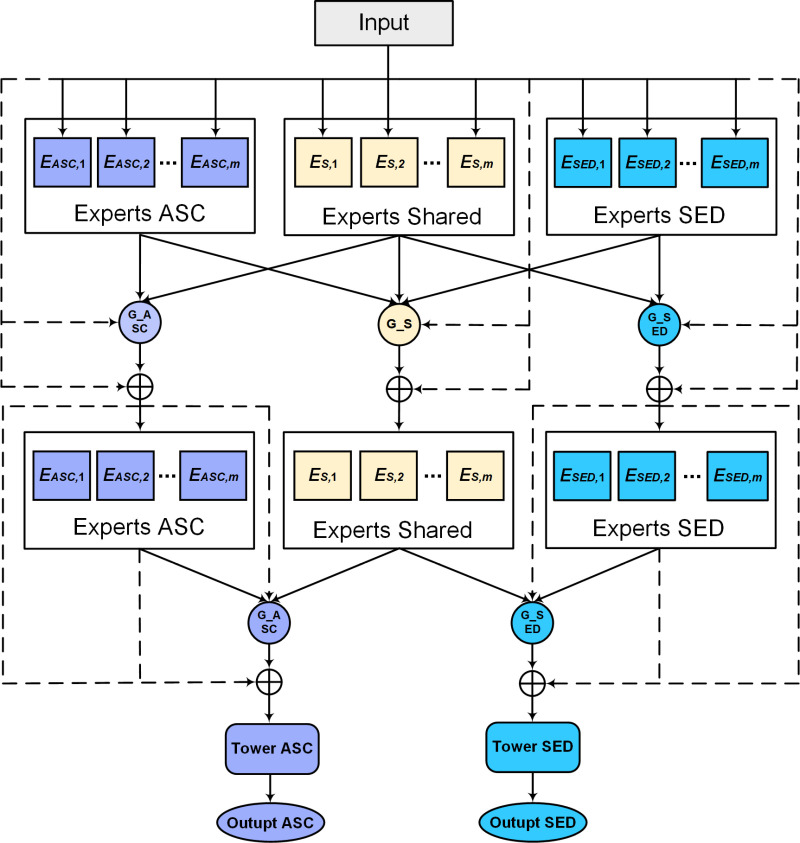
Architecture of proposed MTL-based method.

Conventional MTL methods often lack the flow of information between tasks, and it is difficult to balance the complex interrelationships between SED and ASC. To solve this problem, this study proposes a novel MTL network, as illustrated in Fig 2. The network consists of the R-MFE framework, shared experts, and task-specific experts. In addition, the number of shared experts, task-specific experts and expert units in the R-MFE framework can be flexibly adjusted as required.

The parameter settings in this study are based on the optimal results obtained from multiple experiments. Specifically, five shared experts were established, with each network comprising three layers of MS-conv, aiming at extracting common feature information of sound events and scenes. Meanwhile, five task-specific experts were configured for SED and ASC, respectively. Each task experts employ the same design architecture, which includes one layer of MS-conv and two layers of D-LKAC blocks. The subsequent subsections provide detailed descriptions of each block.

### 3.1 R-MFE framework

The R-MFE framework employs a multi-level feature extraction technique to model interactions between experts. This approach starts with extracting information from the lower-level expert networks and progressively separating task-specific parameters at higher levels. The framework includes two layers of feature extraction network and task-specific tower structure with SED and ASC at the top. The bottom layer of the extraction network processes raw audio features directly, while the second layer handles output from various gating units in the lower network. This study have designed task-specific tower networks at the model’s top. These networks primarily consist of a sequence of linear layers responsible for task categorization. Furthermore, to maximize the flexibility of knowledge sharing, residual connections were introduced to model sharing and task-specific learning separately. Each layer of the R-MFE framework’s extraction network comprises SED task experts, ASC task experts, and shared experts responsible for information sharing. The shared and task-specific expert modules are distinctly separated, with each module consisting of multiple subnetworks, and the number of expert units in each module can be adjusted as a hyperparameter.

In the R-MFE framework, shared and task experts selectively fuse features through the gating unit. The gating unit uses the input as a selector and employs the softmax as the activation function to compute the weighted sum of these chosen vectors, constituting the expert networks’ output. The gating unit’s architecture is built upon a feedforward network, and its output for task *z* can be expressed as:


$$h^{z}\left(x\right)=w^{z}\left(x\right)v^{z}\left(x\right)$$ (4)

where *x* denotes the input feature, $w^{z}(x)$ represents the weighting function, and $v^{z}(x)$ is the matrix consisting of vectors from shared and specific experts for task *z*. The weighted vector of task *z* is computed by linear transformation and SoftMax, expressed as follows:


$$w^{z}\left(x\right)=\text{Softmax}\left(W_{g}^{z}x\right)$$ (5)

where $W_{g}^{z}\in R^{\left(n_{z}+n_{s}\right)\times d}$ denotes the parameter matrix, with *d* as the dimensionality of input features, $n_{z}$ as the number of experts for task *z*, and $n_{s}$ indicates the number of shared experts. Finally, the prediction result for task *z* is denoted as:


$$y^{z}\left(x\right)=t^{z}\left(h^{z}\left(x\right)\right)$$ (6)

Where $t^{z}$ is the tower network for task *z*.

### 3.2 Multi-scale convolution

**Fig 3:**
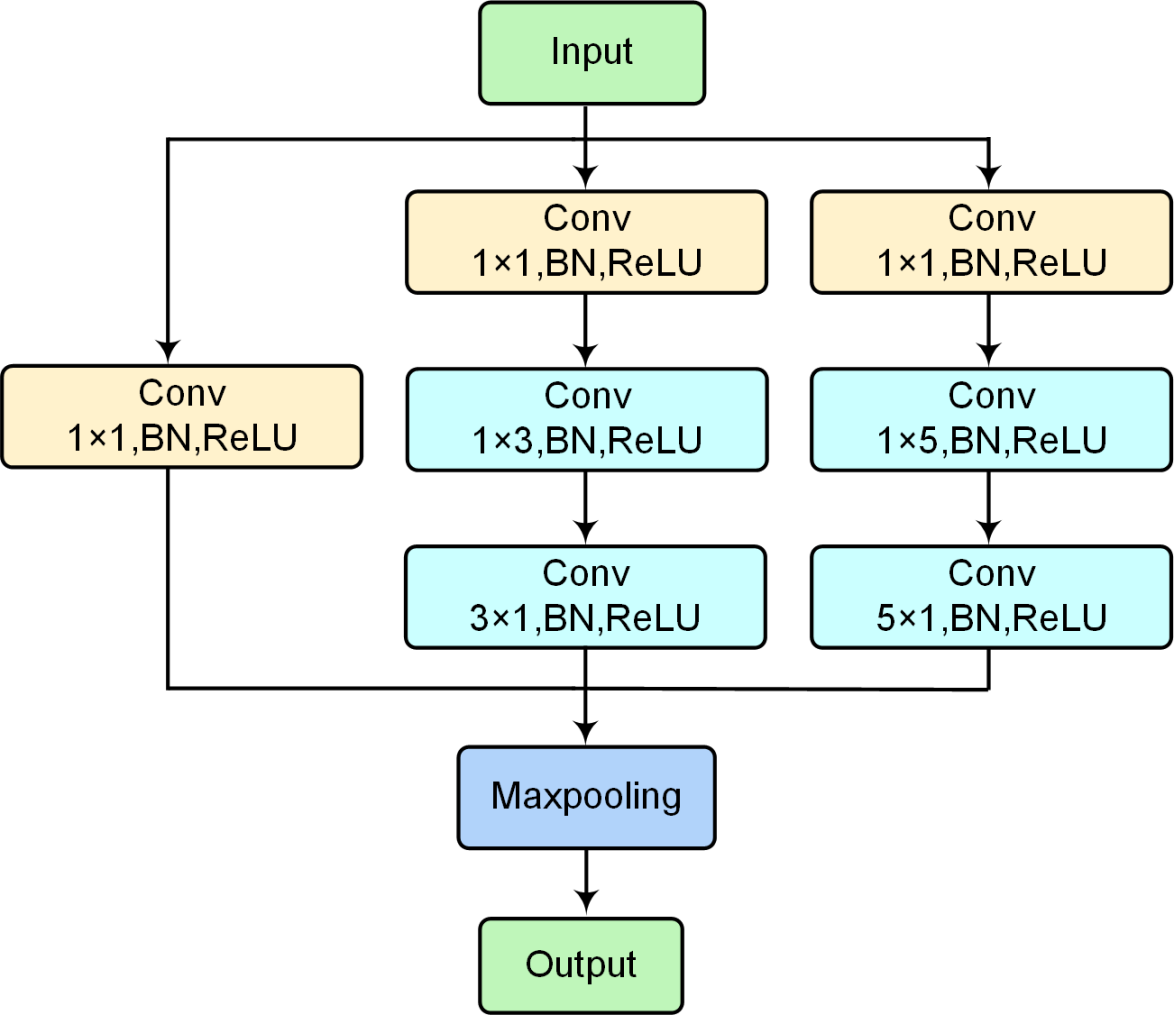
Architecture of the MS-conv module.

The detailed structure of the multi-scale convolution (MS-conv) module is illustrated in Fig 3. This MS-conv module utilizes three sets of parallel convolutions to extract features from log-mel spectra. The convolutions of each scale complement each other, enhancing the model’s ability to capture diverse features. Furthermore, the traditional n × n convolutions are substituted with 1×n and n×1 convolutions to save computational time, where n denotes the convolution kernel size.

In the MS-conv block, the first set of parallel CNN branches consists of a single 1×1 convolutional layer. The second set of parallel CNN branches comprises three convolutional layers with kernel sizes of 1×1, 1×3, and 3×1, respectively. Similarly, the third set of parallel CNN branches contains three convolutional layers with kernel sizes of 1×1, 1×5, and 5×1. Each set of parallel convolutional structures undergoes batch normalization after each convolutional operation to normalize the inputs, followed by applying the ReLU activation function for nonlinear transformation. Finally, the outputs from these parallel convolution groups converge at the maxpooling layer for downsampling, which aims to reduce feature dimensions and enhance the model’s computational efficiency.

### 3.3 Deformable large kernel attention convolution

#### 3.3.1 Overall architecture.

**Fig 4:**
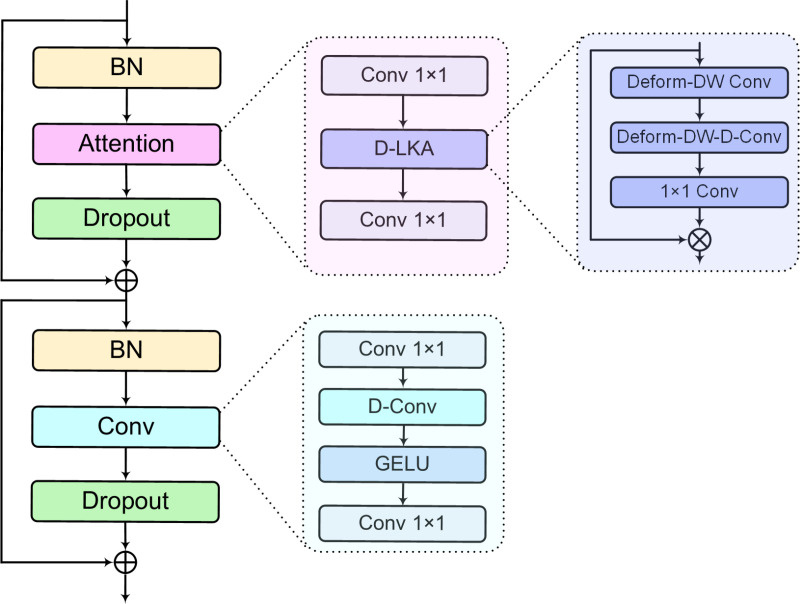
Architecture of D-LKAC block and its components.

Inspired by references [45–48], this study designed a deformable large kernel attention convolution (D-LKAC) block, as depicted in Fig 4. The block comprises a deformable large kernel attention (D-LKA) module, a convolution module, and residual connections. The D-LKA module is an attention mechanism combined with deformable convolution [49], playing an important role. Similar to a self-attention mechanism, it can extract global feature information, effectively overcoming CNN’s limits in processing global information. The convolution module includes a set of symmetrical 1×1 convolution layers that adjust the channel count and depth convolution layers that extract local feature information. The design reduces computational complexity and enhances overall computational efficiency.

Compared to methods just relying on CNNs and Transformer [50], the block merges the benefits of attention and convolution, which can simultaneously capture the global and local features. Unlike the widely used Conformer [45] model, our approach can capture more diverse and rich relevant information than conventional attention mechanisms by dynamically focusing on adjacent time-frequency bands. Furthermore, the residual connections improve the information flow between the network’s shallow and deep layers, reducing information loss during transmission.

#### 3.3.2 Deformable large kernel attention.

Inspired by earlier research in image classification and detection [51,52], this study designed the D-LKA module, as illustrated in purple in Fig 4. The D-LKA module incorporates deformable convolution with depth-wise convolution, depth-wise dilation convolution, and 1×1 convolution, achieving the capability to capture global features with relatively low computational cost and fewer parameters. Specifically, the D-LKA module decomposes standard K×K convolution into three essential steps. Firstly, it gathers local information from the feature map using a (2d−1)×(2d−1) deformable depth-wise convolution (Deform-DW Conv). Secondly, it utilizes a deformable depth-wise dilation convolution (Deform-DW-D-Conv) with a dilation rate of d and dimensions [K/d]×[K/d] to expand the receptive field, thereby capturing long-range dependencies information without adding additional parameters. Finally, it captures channel-wise relationships by a 1×1 convolution.


Attention=Conv1×1(DDW-D-Conv(DDW-Conv(F))) (7)

Output=Attention⊗F (8)

## 4. Experiments

### 4.1 Experimental conditions

#### 4.1.1 Dataset and evaluation metrics.

This study conducted experiments with the TUT Sound Events 2016/2017 and TUT Acoustic Scenes 2016/2017 datasets [53,54]. The dataset contains audio recordings from four distinct acoustic environments: “city center,” “home,” “office,” and “residential area,” totaling 266 minutes. Detailed information about the dataset is available in [55]. The audio recordings cover 25 categories of sound events, each tightly linked to its corresponding acoustic environment. The relationship indicating the presence or absence of these events in the scenes is detailed in Table 1. “1” represents presence, and “0” represents absence.

This study uses Macro-Fscore, Micro-Fscore and Error Rate as evaluation metrics. The Macro-Fscore is computed by aggregating scores across all classes, whereas the Micro-Fscore is obtained by calculating the F-score for each class individually and then averaging these scores. The F-score is computed as:


$$\begin{array}{c} F\text{-score}=\frac{2\cdot \text{Precision}\cdot \text{Recall}}{\text{Precision}+\text{Recall}} \end{array}$$ (9)

Precision and Recall are calculated as follows:


$$\begin{array}{c} Precision=\frac{TP}{TP=FP} \end{array}$$ (10)

$$\begin{array}{c} \begin{array}{c} \;\\ \text{Recall}=\frac{TP}{TP+FN} \end{array} \end{array}$$ (11)

where *TP*, *FP*, and *FN* represent the total number of true positive, false positive, and false negative sound segments, respectively, over all time intervals and acoustic events.

The Error Rate (ER) is calculated as:


$$\begin{array}{c} ER=\frac{\sum_{k=1}^{K}\;S(k)+\sum_{k=1}^{K}\;D(k)+\sum_{k=1}^{K}\;I(k)}{\sum_{k=1}^{K}\;N(k)} \end{array}$$ (12)

where substitution errors *S*(*k*), deletion errors *D*(*k*), and insertion errors *I*(*k*) and are computed as follows:


$$\begin{array}{c} S(k)=\min\left(FN(k),FP(k)\right) \end{array}$$ (13)

$$\begin{array}{c} D(k)=\max\left(0,FN(k)-FP(k)\right) \end{array}$$ (14)

$$\begin{array}{c} I(k)=\max\left(0,FP(k)-FN(k)\right) \end{array}$$ (15)

Here, *k* denotes the time frame index, and *N(k)* indicates the number of sound events present in time frame *k*.

#### 4.1.2 Training setup.

This study employed 64-dimensional log-mel energies as input features, which were calculated for each 40ms timeframe with a 50% overlap. All experiments were conducted on the 3090 GPU. This study applied dropout regularization with a dropout rate of 0.15 after each neural network layer to improve the model’s generalization ability. Furthermore, this study employs an adaptive thresholding method [56] tuned for each sound event to enhance the precision of the SED task by predicting the presence of sound events. Table 2 details the hyper-parameter settings.

**Table 2:** Experimental conditions.

Acoustic feature	Log-mel energy
**Dim**	64
**Frame length/ shift**	40 ms/20 ms
**window**	Hamming
**Optimizer**	Adam
**threshold**	0.4~0.6

### 4.2 Experimental results

#### 4.2.1 Overall performances.

This study evaluates the performance of single-task and MTL approaches in SED and ASC using varying weighting coefficients. To focus on the SED task, the weight λ_SED_ for SED was adjusted to be higher than λ_ASC_ for ASC. Moreover, to ensure a fair comparison, we trained the SED and ASC tasks independently on a network that was consistent with the MTL architecture. The term “Single SED” refers to the network that combines the shared with SED, while “Single ASC” refers to the network that combines the shared with ASC.

Table 3 shows the comparison results between single task and conventional MTL methods. Experimental results demonstrate that MTL methods significantly improve the F-scores of SED and ASC tasks compared to single-task methods. Specifically, when λ_SED_ is set to 0.9 and λ_ASC_ to 0.1, conventional MTL methods achieved increases of 2.43% in Micro-Fscore and 0.89% in Macro-Fscore of the SED task. The findings suggest that sound events and scenes mutually enhance. Particularly in SED tasks, information from acoustic scenes can enhance detection performance. However, conventional MTL methods have certain limitations compared with our proposed method.

**Table 3:** Performance of conventional methods for SED and ASC.

Method (λ_sed_, λ_asc_)	Scene		Event		
	Micro-Fscore(%)	Macro-Fscore(%)	Micro-Fscore(%)	Macro-Fscore(%)	ER
**Single ASC**	97.08	97.36	—	—	—
**Single SED**	—	—	46.63	13.78	0.711
**MTL (0.9, 0.1)**	95.96	96.37	**49.06**	**14.67**	**0.674**
**MTL (0.8, 0.2)**	**97.47**	**97.72**	41.77	10.38	0.723
**MTL (0.7, 0.3)**	97.09	97.38	40.56	9.75	0.725
**MTL (0.6, 0.4)**	96.34	97.61	40.59	9.13	0.732

Table 4 shows the comparison results between our proposed MTL and single-task methods. The results demonstrate that our MTL method achieves optimal performance when λ_SED_ is set to 0.9 and λ_ASC_ to 0.1. Compared to conventional MTL models, our method shows increases of 7.87% and 21.8% in the Micro-Fscore and Macro-Fscore for the SED task, with particularly significant improvements in Macro-Fscore. The Macro-Fscore is calculated by averaging the F-scores of each sound event, which reflects the model’s balance in recognizing various events. Experimental results validate the efficacy of our MTL method. By clearly distinguishing shared and task-specific experts in the conventional MTL framework, the proposed method minimizes interference from harmful parameters between tasks. Furthermore, the multi-level feature extraction strategy facilitates the transfer of information between tasks and improves the overall performance.

**Table 4:** Performance of the proposed method for SED and ASC.

Method (λsed, λasc)	Scene		Event		
	Micro-Fscore(%)	Macro- Fscore(%)	Micro-Fscore(%)	Macro- Fscore(%)	ER
**Single ASC**	94.32	94.80	—	—	—
**Single SED**	—	—	47.74	26.02	0.789
**MTL (0.9, 0.1)**	95.58	95.96	56.93	36.47	0.645
**MTL (0.8, 0.2)**	94.44	94.99	51.62	31.59	0.696
**MTL (0.7, 0.3)**	93.18	93.73	43.66	24.96	0.751
**MTL (0.6, 0.4)**	95.83	96.10	37.47	20.28	0.812

#### 4.2.2 Detailed investigation of SED.

To further investigate the performance of the SED, we evaluated the classification results of 25 different types of sound events, as detailed in Table 5. The results show that our proposed MTL approach improves the F-scores for many sound events, notably “(object) banging,” “ car,” and “people talking.” The improvement is attributed to the close connection between these sound events and the specific scene “residential area”, as shown in Table 1. This finding suggests that the proposed MTL method can effectively mine and utilize the strong correlations between acoustic scenes and events.

**Table 5:** Average segment-based F-scores for event sound events.

Event				(object) banging		(object) impact		(object) rustling		(object) snapping		(object) squeaking		Bird singing		brakes squeaking		breathing			car	children		cupboard		cutlery	
**Single SED**		**F-score(%)**		42.86		51.07		0.00		62.91		0.00		54.71		**8.23**		0.31			64.32	2.92		64.43		40.39	
		**ER**		1.21		1.31		1.12		0.71		1.00		0.95		1.44		1.09			0.75	1.06		0.79		1.34	
**Conventional methods**		**F-score(%)**		50.69		51.25		0.00		63.64		0.00		51.38		6.71		0.00			63.02	0.35		79.09		38.69	
		**ER**		0.93		1.17		1.00		0.69		1.00		0.81		1.00		1.00			0.88	**1.00**		0.39		0.82	
**Proposed** **(λ**_**SED**_ **=0.9)**		**F-score(%)**		**57.52**		**56.47**		0.13		**76.59**		0.00		**62.48**		7.71		0.00			**66.35**	**6.66**		**83.36**		**51.48**	
		**ER**		**0.86**		1.11		1.01		**0.46**		1.00		**0.77**		**1.01**		1.00			**0.74**	1.01		**0.33**		**0.77**	
**Event**			**dishes**		**drawer**		**fan**		**glass** **jingling**		**keyboard typing**		**large vehicle**		**mouse clicking**		**mouse wheeling**		**people talking**	**people walking**			**Washing dishes**		**Water** **tap running**		**wind blow** **ing**
Single SED	F-score(%)		25.40		17.71		0.00		0.00		33.61		0.52		39.95		3.37		46.92	32.34			29.87		28.57		0.00
	**ER**		1.49		0.99		1.00		1.00		**1.25**		1.27		1.66		1.36		1.01	1.32			1.10		1.29		1.00
**Conventional methods**	**F-score(%)**		71.08		32.68		0.00		0.00		42.83		0.00		37.65		15.89		54.08	36.27			49.55		44.79		0.00
	**ER**		0.52		0.83		1.00		1.00		1.27		1.05		1.06		**1.03**		0.92	0.99			0.79		0.97		1.00
**Proposed** **(λ**_**SED**_ **=0.9)**	**F-score(%)**		**75.17**		**47.04**		0.00		0.00		**45.83**		0.00		38.56		**26.77**		**58.94**	**44.91**			**58.24**		**47.56**		0.00
	**ER**		**0.45**		**0.78**		1.00		1.00		1.26		**1.04**		**1.27**		1.06		**0.79**	**0.96**			**0.73**		**0.96**		1.00

Due to data imbalances, some sound class such as “fan”, “rustling”, “squeaking” and “breathing” have relatively few training samples, resulting in limited performance improvements for these classes. To address this issue, future work will involve several enhancements. For example, data augmentation techniques such as noise addition, time stretching, and frequency shifting will be used to increase the representation of these categories in the training data. Additionally, weighted loss functions will be explored, assigning higher weights to these underrepresented classes during training to improve the learning process for imbalanced categories.

In summary, the MTL approach surpasses single-task and conventional methods, achieving higher F-scores and reduced ER in various sound events. The findings emphasize the importance of acoustic scenes to enhance the accuracy of SED in acoustic environment analysis.

#### 4.2.3 Ablation studies.

In this section, a series of ablation studies were conducted to evaluate the effectiveness of each module in our proposed MTL network. Table 6 shows the detailed analysis results. Using the conventional MTL model described in Section 2 as the baseline, we sequentially evaluated the contributions of the P_MFE framework, MS-conv block, and D-LKAC block in enhancing SED performance.

**Table 6:** Comparison of ablation experimental results.

Method	Micro-Fscore(%)	Macro- Fscore(%)
**Baseline**	49.06	14.67
**P_MFE**	49.57	30.12
**P_MFE+MS-conv**	52.57	33.14
**P_MFE+DLKAC**	53.19	33.41
**Proposed**	**56.93**	**36.47**

Experimental results indicate that the introduction of the P_MFE framework enhances the SED task’s performance, increasing the Micro-Fscore by 0.51% and the Macro-Fscore by 15.45%, respectively. It is worth noting that even without MS-conv and D-LKAC block, the P_MFE framework still outperforms conventional MTL Methods. This demonstrates that the P_MFE framework effectively sustains ongoing interactions and information flow across tasks, and deeply explores more complex relationships between acoustic scenes and events, promoting joint optimization. With the introduction of the MS-conv block, SED performance further increased by 3%, highlighting the crucial role of the MS-conv in capturing subtle features of audio data. The addition of the D-LKAC module enhanced performance by 3.62%, showing its strong dynamic adaptability. This module efficiently captures both global and local features from audio sequences, thereby enhancing SED’s performance.

#### 4.2.4 Comparison with other methods.

To assess the superiority of the proposed MTL method, this study conducted comparisons with existing SED and ASC joint analysis methods. Table 7 shows the detailed comparison results. The compared methods follow the MTL model, which is based on hard parameter-sharing described in Section 2. Section 1 provides detailed descriptions of the design principles and characteristics of each method.

**Table 7:** Comparison with state-of-the-art methods in SED.

#	Reference	F-score(%)	Params(KB)
**1**	[36]	42.79	—
**2**	[37]	44.27	—
**3**	[31]	44.64	—
**4**	[38]	46.07	—
**5**	[57]	50.52	—
**6**	[30]	49.57	—
**7**	[29]	49.82	—
**8**	[58]	46.98	448.88
**9**	[59]	43.06	223.72
**10**	[60]	47.13	572.59
	**Proposed**	**56.93**	**394.85**

Experiments demonstrate that our proposed MTL approach surpasses current methods on the TUT 2016/2017 datasets. Specifically, the F-scores for the SED task reached 56.93%, which is 6.44 percentage points higher than the previous best model [34]. This improvement is attributed to the proposed MTL model’s ability to effectively balance complex interactions of SED and ASC tasks, maintaining information throughout the training process. Furthermore, the introduction of MS-conv and D-LKAC blocks enables the model to capture more subtle event features. Experimental results prove the proposed MTL model’s superiority, demonstrating its excellent coordination and generalization capabilities in capturing complex interactions between SED and ASC tasks.

## 5. Conclusion

This study proposes a multitask network based on the R-MFE framework, aimed at exploring the relationship between acoustic scenes and events, and utilizing scene information to enhance the performance of sound event detection. The proposed method overcomes the limitations of information interaction and flow between tasks in conventional MTL approaches and effectively balances the complex interrelationships between SED and ASC tasks. Moreover, this study introduces the D-LKAC attention module, which captures both global and local contextual features, and extracts richer feature information by dynamically focusing on adjacent time-frequency bands compared to conventional attention mechanisms. To further optimize the performance of the SED task, the MS-conv module is designed to capture audio detail features from multiple dimensions. We conducted experiments using the TUT 2016/2017 dataset to evaluate the performance of and SED. Experimental results indicate that the proposed method outperforms single-task learning and conventional MTL approaches, achieving a 6.44% improvement in F-score compared to current state-of-the-art methods. These experiments confirm the effectiveness of the proposed MTL method, and show that scene information significantly enhances SED performance.
